# Probabilistic Modelling of Fracture Toughness of Composites with Discontinuous Reinforcement

**DOI:** 10.3390/ma16082962

**Published:** 2023-04-07

**Authors:** Grzegorz Mieczkowski, Tadeusz Szymczak, Dariusz Szpica, Andrzej Borawski

**Affiliations:** 1 Faculty of Mechanical Engineering, Bialystok University of Technology, 45C Wiejska Str., 15-351 Bialystok, Poland; 2 Department of Vehicle Type-Approval & Testing, Motor Transport Institute, 80 Jagiellonska Str., 03-301 Warsaw, Poland

**Keywords:** composites, effective fracture toughness, probabilistic modelling, Weibull distribution

## Abstract

The results presented in the paper are related to the prediction of the effective fracture toughness of particulate composites (*K*_IC*eff*_). *K*_IC*eff*_ was determined using a probabilistic model supported by a cumulative probability function qualitatively following the Weibull distribution. Using this approach, it was possible to model two-phase composites with an arbitrarily defined volume fraction of each phase. The predicted value of the effective fracture toughness of the composite was determined based on the mechanical parameter of the reinforcement (fracture toughness), matrix (fracture toughness, Young’s modulus, yield stress), and composite (Young’s modulus, yield stress). The proposed method was validated: the determined fracture toughness of the selected composites was in accordance with the experimental data (the authors’ tests and literature data). In addition, the obtained results were compared with data captured by means of the rule of mixtures (ROM). It was found that the prediction of *K*_IC*eff*_ using the ROM was subject to a significant error. Moreover, a study of the effect of averaging the elastic–plastic parameters of the composite, on *K*_IC*eff*_, was performed. The results showed that if the yield stress of the composite increased, a decrease in its fracture toughness was noticed, which is in line with the literature reports. Furthermore, it was noted that an increase in the Young’s modulus of the composite affected *K*_IC*eff*_ in the same way as a change in its yield stress.

## 1. Introduction

Composites are currently used in many key areas of industry. They can be found in the following branches: automotive [1], aerospace [2], military [3], and civil engineering [4]. Compared to commonly used materials, they exhibit better thermal [5], electrical [6], tribological, or mechanical [7] properties. This is connected with the best features of the matrix such as: ductility, fracture toughness, low specific weight, and reinforcement parameters as follows: high values of ultimate strength and the elastic modulus, as well as a good wear resistance [8,9].

There is no doubt that the final features of a composite are most influenced by the mechanical properties of its individual components. However, the manufacturing technology [7,10], as well as the distribution, geometry, and size of the reinforcement particles [11,12] are also important. It should be noted that an improvement in the selected mechanical parameter is often associated with a decrease in another parameter. In the case of metallic matrix composites reinforced with ceramics, if the volume fraction of the reinforcement increases, the yield stress and ultimate tensile strength increase, but the fracture toughness decreases [13]. Thus, there is a well-founded need to determine the effective mechanical properties, including the fracture toughness (*K*_IC*eff*_), of newly developed composite materials.

The most-reliable manner for determining the effective fracture toughness of composites is related to experiments. For metal matrix composites (MMCs), with the single reinforcement by SiC or Al_2_O_3_ particles, the experimental results were presented by the authors of the papers [13,14,15,16]. The disadvantages of this approach are that it is time-consuming and laborious.

As an alternative to experiments, analytical or numerical methods can be used. The finite-element method (FEM) is very often applied to model many physical problems, with the FEM supporting the aim of the strength of materials [17,18,19], friction [20,21], heat flow [22,23], and fluid flow [24,25,26,27], as well as piezoelectricity [28,29]. Using this method, it is also possible to predict the effective fracture toughness of particle-reinforced composites. However, the prediction of *K*_IC*eff*_ using the FEM, as with analytical models, is complicated and difficult. This is due to the fact that modelling the fracture process is limited by a crack growing in various material components, i.e., the crack can be located in the matrix, the inclusion, or at the interface. Nevertheless, some interesting results for *K*_IC*eff*_ can be noticed in [30,31].

Numerous analytical models have been developed to predict the *K*_IC*eff*_ of composite materials. Some of them [32,33,34] use a local approach with the following relationships—*K*_IC*eff*_ = *f*(*R*, *L*, *K_Icm_*, *K_Ici_*), where *R* is the radius of the reinforcement particles, *L* is the distance between the particles, and *K_Icm_* and *K_Ici_* are the fracture toughness of the matrix and reinforcement, respectively. This gives an acceptable prediction of the results, but for a certain range of the *R/L* ratio [35]. Statistical/probabilistic models can be considered as slightly different local approaches to predicting *K*_IC*eff*_. For this case, these methods were firstly applied to homogeneous materials with respect to the occurrence of different fracture mechanisms—brittle, ductile, or their combination [36,37]. Due the fact that, in composites, fracturing can occur in the matrix, reinforcement, or at the interface (different fracture mechanisms), this approach was adopted to predict their *K*_IC*eff*_. In the papers [38,39], probabilistic models based on local parameters such as the reinforcement particle size and distribution in the composite were developed to enable the prediction of the effective fracture toughness.

Nevertheless, it is worth noticing that the authors proposed a probabilistic model based on the global parameters of the composite—the effective Young’s modulus (*E_c_*) and yield stress (*σ_yc_*). It is obvious that the value of these parameters will be affected by the shape, size, and distribution of the reinforcement [40], so that the microstructure of the composite was indirectly taken into account in the proposed model. Thus, the developed model is more versatile and can be used to predict the effective fracture toughness of composites with a discontinuous reinforcement of any form such as particles, short fibres, or whiskers. The probabilistic model, described in Section 2, adopted the cumulative probability function for fracture as a Weibull distribution. The experimental validation (the authors’ tests and literature data) of the proposed approach is presented in Section 3.

The authors’ effort was also focused on the variations of the value of *K*_IC*eff*_ due to changes in the values of the global parameters of the composite (*E_c_*, *σ_yc_*) and the mechanical parameters of the reinforcement materials (fracture toughness (*K_Ici_*)), matrix (Young’s modulus (*E_m_*), yield stress (*σ_ym_*)). The results are detailed in Section 4.

## 2. Materials and Methods

### 2.1. Probabilistic Model for Determining Effective Fracture Toughness

Weibull was the first to develop a statistical theory for the brittle fracture of homogeneous materials [41,42]. His basic assumption was the hypothesis that total failure is controlled primarily by the weaker elements and that the strength of the least strong element is very low. Using the empirical arguments that were necessary to obtain a simple and accurate fit to his own experimental data, he developed a formula for the so-called Weibull fracture probability distribution (function). This distribution, like any other distribution, has its advantages (simplicity, flexibility, applicability to small datasets) and limitations (e.g., monotonicity) [43].

Based on Weibull’s studies, the authors of the paper [44] developed a statistical model to predict the strength of homogeneous materials. The model assumed that the cumulative fracture probability function depends on the stress state, determined in space, defined as the volume of the material. The loss of material cohesion occurs when the level of cumulative stresses, determined according to the adopted specific risk of fracture function (RFF), reaches a fixed limit. It should be mentioned that the genesis of the RFF is related to the statistical theory of fracture [41] and describes the risk of the loss of cohesion of a material depending on its stress state and other factors affecting its strength. It is based on the assumption that there are a certain number of defects or inhomogeneities in each material sample that represent potential locations for the initiation of the fracture process. It can be used to determine safe loading levels and to develop criteria/models for assessing/estimating the rheological properties of a material.

The proposed probabilistic model was developed based on the statistical theory of fracture, suggested by the authors of the paper [44]. The generalisation of the fracture model presented in the paper [44] to the case of a composite whose individual material fractions are characterised by different fracture toughness, which depends on the local yield stress, has enabled formulating the cumulative fracture probability function *F*(*K*_I_), describing the scatter of the fracture toughness of the composite in the following form:

(1) $$F\left(K_{I}\right)=1-\exp\left\{-\frac{1}{\sigma_{ym}}\int_{\sigma_{y}}\phi \left[K_{I}\left(\sigma_{y}\right)\right]d\sigma_{y}\right\},$$

where: *K*_I_—maximum stress transfer capacity of the material without crack initiation, *K*_I_(*σ_y_*) ≤ *K*_I_—local fracture toughness, *σ_ym_*—yield stress of the matrix, *σ_y_*—local yield stress, *σL_y_*—measurable set, *ϕ*[*K*_I_(*σ_y_*)]—the specific risk of fracture function RFF.

In the approach presented here, fracture toughness was assumed to be locally variable, and the material was treated as a set of elements (measurable set) *σL_y_*, for which a local parameter *K*_I_(*σ_y_*) was assigned. In turn, the function *ϕ*[*K*_I_(*σ_y_*)] describes the risk of losing the material’s ability to resist a propagating crack.

When the averaged properties of a composite are known, it can be treated as a homogeneous material. This allows simpler mathematical models to be used to study its mechanical properties. Thus, when treating the composite as a homogeneous material for which the effective yield stress is known, the set *σL_y_* becomes a one-element set for which *K*_I_(*σ_y_*) = *K*_I_, and Equation (1) takes the following form:

(2) $$F\left(K_{I}\right)=1-\exp\left\{-\frac{\sigma_{yc}}{\sigma_{ym}}\phi \left[K_{I}\right]\right\},$$

where: *σ_yc_ = σ_y_*—effective yield stress of the composite.

Assuming that a Weibull function is used as the specific risk of fracture function, with the amendment proposed in the paper [45], the formula *ϕ*[*K*_I_] can be written in the following form:

(3) $$\phi \left[K_{I}\right]=\left\{\begin{array}{l} {\left[\left(K_{I}-K_{\mathrm{Imin}}\right)/K_{o}\right]}^{m}\;\;\;\;\;\;\;\;\;\;\;K_{I}\geq K_{\mathrm{Imin}}\;\;\;\\ 0\;\;\;\;\;\;\;\;\;\;\;\;\;\;\;\;\;\;\;\;\;\;\;\;\;\;\;\;\;\;\;\;\;K_{I}<K_{\mathrm{Imin}} \end{array}\right.,$$

where *K_o_*—normalising coefficient, *m*—a shape parameter, *K*_Imin_—minimum value of *K*_I_, e.g., the fracture toughness of the matrix material (*K*_Imin_ = *K_Icm_*, *K_Icm_* < *K_Ici_*) or reinforcement (*K*_Imin_ = *K_Ici_*, *K_Icm_* > *K_Ici_*); when exceeded, the fracture process can be initiated.

The amendment [45] is intended to allow the function *ϕ*[*K*_I_] to meet the following necessary conditions—to be positive, non-decreasing, and vanishing at a value that is not necessarily equal to 0.

Both *K_o_* and *m* depend on the properties of the materials and the method of manufacturing. *K_o_* is set so that when a fracture is potentially initiated, *F*(*K*_I_) is a minimum of 50% [46]. The shape parameter, *m*, is a measure of the dispersion of fracture toughness [47]. Large values of *m* indicate homogeneity, whereas small values represent high dispersion.

In most existing probabilistic models, the values of *K_o_* and *m* are determined by fitting a Weibull distribution function to the experimental data, using, for example, linear regression [39]. In the paper presented here, the normalising factor was assumed to be calculated using the following formula (the form of the formula was adopted arbitrarily and satisfies the previously mentioned condition *F*(*K*_I_) ≈ 50%):

(4) $$K_{o}=\left(1+\left(1-V_{i}\right){\left(\frac{E_{m}}{E_{c}}\right)}^{2}\right)\left(\frac{K_{Icm}+K_{Ici}}{2}\right)-K_{I\min},$$

where: *V_i_*—volume fraction of reinforcement, *E_c_*, *E_m_*—Young’s modulus of the composite and matrix material, respectively.

As for the parameter *m*, its value was arbitrarily assumed to be constant and equal to *m* = 4.

Equation (2), by analogy with reliability theory, can be presented as below [43]:

(5) $$F\left(K_{I}\right)=1-R(K_{I}),$$

where *R*(*K*_I_) is a reliability function.

Taking into account Formulas (3) and (4), the reliability function can be expressed in the following form:

(6) $$R\left(K_{I}\right)=\exp\left\{-\frac{\sigma_{yc}}{\sigma_{ym}}{\left(\frac{K_{I}-K_{I\min}}{\left(1+\left(1-V_{i}\right){\left(\frac{E_{m}}{E_{c}}\right)}^{2}\right)\left(\frac{K_{Icm}+K_{Ici}}{2}\right)-K_{I\min}}\right)}^{4}\right\}\;\;$$

Using the analogy of reliability theory, the effective fracture toughness of the composite can be considered as the so-called mean time to failure (MTTF) and determined using the following formula [43]:

(7) $$K_{ICeff}=\int_{0}^{\infty }R(K_{I})dK_{I}$$

Undoubtedly, the fracture toughness of a composite depends largely on its microstructure. The size, shape, and distribution of the reinforcement phase affect the initiation and propagation of microcracks. In the proposed approach, the above factors were not taken into account directly. They were taken into account indirectly: namely, in the cumulative fracture probability function *F*(*K*_I_), the microstructure of the composite was explicitly determined by its Young’s modulus (*E_c_*) and yield stress (*σ_yc_*). The value of *E_c_* and (*σ_yc_*) depends on the geometry and distribution of the reinforcement [11,12]. The proposed statistical model can be used only for composites with discontinuous reinforcement (particles, short fibres/whiskers) with a random distribution. For composites with continuous reinforcement with a defined fibre orientation, the *F*(*K*_I_) function would need to be modified in the model. The modification should take into account the effect of the fibre orientation on *E_c_* and *σ_yc_*.

### 2.2. Probabilistic Model Validation

The proposed methodology for determining the effective fracture toughness of the composite was verified by comparing the results obtained using it with experimental data. The literature data and the authors’ tests were used for the verification. A comparison of the effective fracture toughness of the analysed composites, determined using the developed probabilistic model, with experimental data is presented in Section 3. Regarding our own experimental research, experimental *K*_ICd_ values and other necessary material parameters (*σ_yc_*, *E_c_*) were determined for the Al44200/Al_2_O_3_ composite (with an aluminium matrix and Al_2_O_3_ fibre reinforcement (Saffil)). The test methodology used to determine the fracture toughness in an offset manner is described below.

#### 2.2.1. Experimental Determination of Fracture Toughness of Al44200/Al_2_O_3_ Composite

As mentioned above, the experimental determination of the fracture toughness of composites has both advantages and disadvantages. A significant advantage is related to capturing the mechanical parameters directly in tests. As for the disadvantages, the time consumption and high costs should be mentioned. Nevertheless, the laboratory stage of determining the mechanical properties of a composite by the average approach is crucial and cannot be omitted in any way. It is, therefore, advisable to pre-determine the structure of the composite, with the desired mechanical–physical properties, by means of other methods (e.g., predictive models) and then carry out laboratory testing of the produced composite.

**Figure 1:**
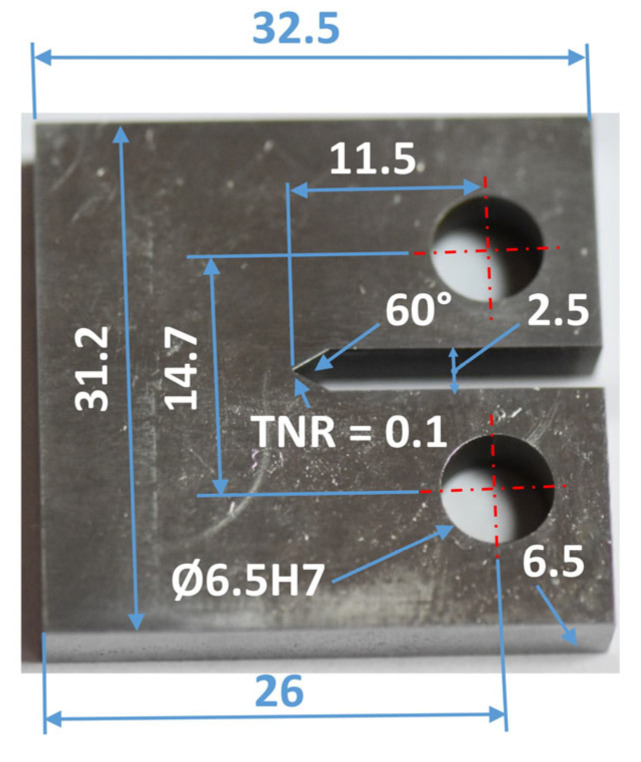
CT mini-specimens used in the fracture toughness tests of the MMC.

**Figure 2:**
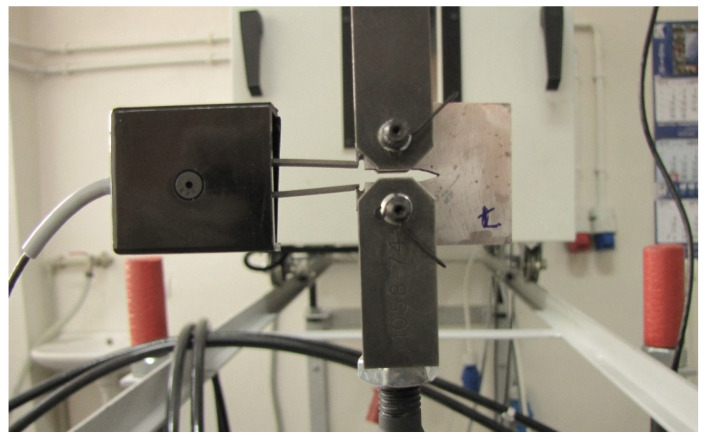
CT mini-specimen made of the 44200 aluminium alloy with the Al_2_O_3_ reinforcement and CTOD Instron extensometer with the gripping system of the 8802 Instron servo-hydraulic testing machine.

In many cases, mechanical tests require the design of appropriate specimen sizes (Figure 1) and fastening systems for mounting in a testing machine (Figure 2). This applies to both basic tests, such as tensile and bending tests, as well as more advanced experiments: the fracture toughness test. In the case of determining the fracture toughness, the European [48], British [49,50], and American [51] standards can be used, because they contain complete information on the geometry of the specimens, their dimensions, and their proportions, including a comparison of the dimensions with the relationship between the fundamental mechanical parameters such as: the yield stress to the elastic limit of the material examined. They cover a minimum value of fatigue pre-crack length and a number of cycles for the fatigue stage. Moreover, they collect equations and graphs for fracture toughness analysis [52]. These approaches were chosen to determine the resistance of the metal matrix composite (MMC) with the 44200 aluminium alloy matrix with the Saffil (Al_2_O_3_) short fibre reinforcement [53,54]. In this case, compact tension (CT) mini-specimens (Figure 1) were used with a special gripping system (Figure 2). Despite CT specimens being able to have different dimensions, they should be examined with respect to the correct results using a conventional material with a known value of the stress intensity factor (SIF) or crack tip opening displacement (CTOD) [53].

The stages for the fracture toughness test from a practical point of view can be collected as follows:

- CT specimen designed according to the requirements of the PN-EN ISO 12737 [48], BS 186, BS EN ISO 12737 [49], ASTM E1820 [50], and ASTM 399 [52] standards;
- CT specimens manufactured by means of electrical discharge machining (EDM);
- Measurements of CT specimens’ dimensions using an optical microscope, covering the width, height, and tip radius of the notch and the holes and their location with respect to the specimen’s opposite side;
- Determination of Young’s modulus and the yield stress (if possible, in the opposite case, the values of these parameters should be approached based on the mechanical parameters of the matrix and reinforcement);
- The fatigue pre-crack stage is recommended to be conducted by means of special software directly intended for this process. If this stage is to be correct, the minimum value of the fatigue crack length should be obtained, i.e., 2.5% W (or 1.3 mm) with the number of cycles between 10^4^ and 10^6^ in the stress ratio range from −1 to +0.1 according to the requirements of the ASTM E1820 standard [51]. The force amplitude should be selected for the elastic state of the material;
- Testing under a monotonic tensile force is recommended to be conducted directly after the fatigue pre-cracking. This should be performed up to a total decohesion of the specimen following a crack growing by means of the macrophotography technique;
- The cracking plane does not deviate more than 10% from the horizontal one as in ASTM E399 [52];
- The determination of the fatigue pre-crack length at five points followed by 0%, 25%, 50%, 75%, and 100% with respect to the width value. Differences between the nearest values collected should be lower than the 10% average value (determined without the crack front length at the specimen edges (ASTM E1820) [51]). Moreover, the difference between the measured fatigue pre-crack lengths on both surfaces should not exceed 15% of the average value from both measurements (ASTM E399) [52];
- The determination of the K_Q_ value in the regime P_max_/P_Q_ < 1.1 (ASTM E1820 [51], ASTM E399 [52]).

Calculate the K_Q_ value (ASTM E1820 [51], ASTM E399 [52]), and check whether these requirements are fulfilled: 2.5 · K^2^_Q_ < B YS^2^, 2.5 · K^2^_Q_ < a YS^2^, then this parameter represents a critical value of the stress intensity factor (SIF).

**Figure 3:**
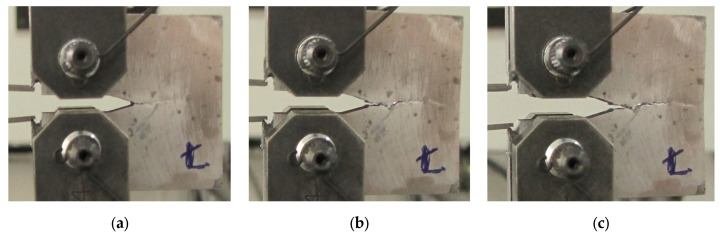
CT mini-specimen made of 44200 aluminium alloy with 15% Al_2_O_3_ reinforcement under monotonic tension: (**a**) the initial stage; (**b**,**c**) the stages before fracture.

**Figure 4:**
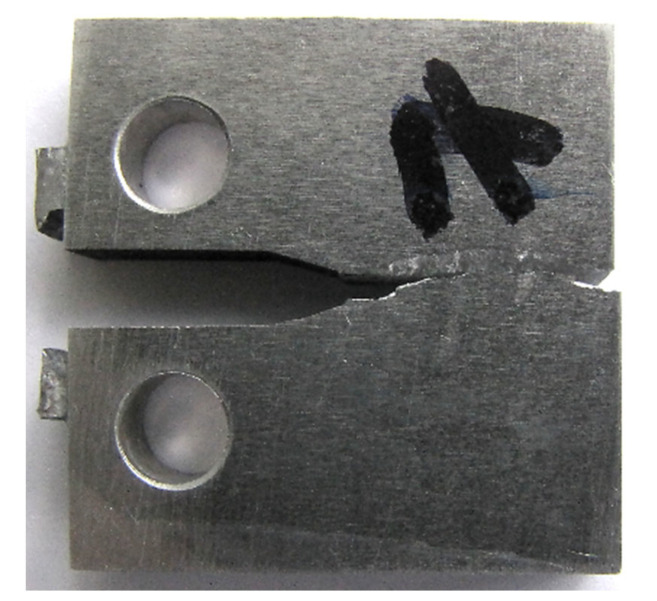
CT mini-specimen made of 44200 aluminium alloy with 15% Al_2_O_3_ reinforcement (Saffil fibres) after the fracture toughness test.

If a composite material is subjected to testing, the development of fatigue cracks under the conditions of monotonic tensile force should be recorded in several stages (Figure 3 and Figure 4). This can be achieved using the macro-photography technique with an additional uniform lightening source if is need. Thanks to this approach, it is possible to observe the course of cracking of the tested material and to make initial conclusions about the quality of the obtained result in order to qualify for further calculations and analyses. This type of procedure was used to determine the fracture toughness of a metal composite with a matrix made of aluminium alloy of the 44200 grade reinforced with Al_2_O_3_ fibres in a percentage range from 10% to 20% [53,54]. For this kind of material fatigue, the regions did not express any typical features observed for conventional metals. The obtained results showed the SIF values in a relatively narrow range, allowing for averaging up to a value of 12 MPa m^0.5^. The determined SIF, yield stress, and Young’s modulus values were used to validate the probabilistic model proposed in this paper.

**Figure 5:**
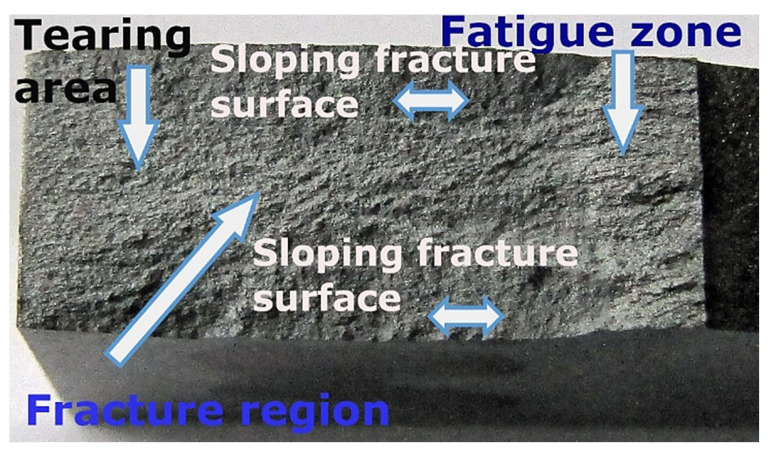
Damage zones of the 44200 aluminium alloy composite with 15% Al_2_O_3_ reinforcement (Saffil fibres) captured in the fatigue pre-cracking and monotonic tensile stages of the fracture toughness test.

**Figure 6:**
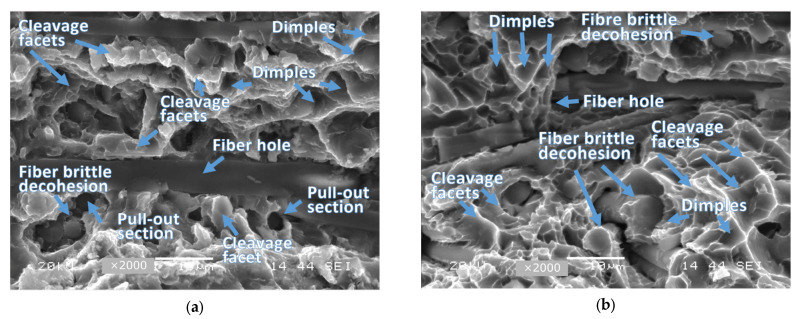
Damage zones of the 44200 aluminium alloy composite reinforced by 15% Al_2_O_3_ (Saffil fibres) at a magnification of ×2000: (**a**) fatigue pre-crack region; (**b**) tearing section.

The macroscopic results of the material tested were taken into account with respect to the following features for fatigue and other regions, i.e., fracture and tearing (Figure 5). Nevertheless, this kind of data did not reveal any significant geometrical features such as: fatigue lines, the dimensions of this region, and the shape of the fatigue pre-crack edge. Generally, it can be concluded that this type of data indicated difficulties in determining the fatigue zone due to the lack of these features. This means this kind of result is different than what is observed in typical structural materials such as steel, covering its high strength grade [55]. Moreover, no differences in the fracturing mechanisms due to the fatigue pre-cracking (Figure 6a) and monotonically increasing tensile force (Figure 6b) were observed. For these cases, the following features can be indicated: cleavage regions, dimples, pull-out zones, fibre brittle decohesion, and fibre holes. This enabled collecting the advantages, disadvantages, important results, and applications for the MMC material considered (Table 1).

## 3. Results

### 3.1. Validation Results and Discussion

Table 2 and Table 3 show the material parameters of the composite, matrix, and reinforcement, including the fracture toughness determined as described in Section 2.2.1 and using Equation (7). Table 3 also provides the value of the cumulative fracture probability *F*(*K*_IC*eff*_) and the upper (8) and lower (9) fracture toughness limits of the composite determined using the rule of mixtures (ROM).

(8) $$K_{u}=K_{Ici}V_{i}+\left(1-V_{i}\right)K_{Icm}$$

(9) $$K_{l}={\left(\frac{V_{i}}{K_{Ici}}+\frac{1-V_{i}}{K_{Icm}}\right)}^{-1}$$

More precise models than the ROM can be found in the literature. In this paper, the use of existing models was abandoned due to their considerable complexity. They often require extensive data such as a detailed description of the microstructure and, often, its spatial/plane modelling and FEM numerical testing.

Analysing the obtained results, one can conclude that the *K*_IC*eff*_ values determined by the developed probabilistic model coincide with the experimental data. The maximum relative error was about 2%.

The *K*_IC*eff*_ values predicted by means of Equation (7) were also compared with available literature data. There are many papers in the literature in which the authors determined the effective fracture toughness of the composites under consideration through experimental studies. Unfortunately, in the majority of the reports, the authors did not provide complete data (yield stress and Young’s modulus of the composite, fracture toughness of individual material phases) to enable tests to be carried out using the proposed probabilistic model. Due to the lack of sufficient comparative data, a small number of samples were used to validate the model. In the work presented here, the experimental results reported in the papers [15,40,59,60,61,62] were used as the comparative data. In addition to the effective fracture toughness of the composites investigated, the authors of the papers also provided most of the other material parameters necessary for prediction using the developed model. The material parameters of the composites analysed and the predicted fracture toughness values are shown in Table 4 and Table 5. The composites considered were characterised by varying microstructures—different matrix and reinforcement materials were used, with different volume fractions of the latter. This ultimately impacted the global parameters (the yield stress and Young’s modulus of the composite) used in the probabilistic model.

Based on the analyses, one can conclude that, for the composites shown in Table 5, as for the Al4420/Al_2_O_3_ composite, the predicted *K*_IC*eff*_ values coincided with the experimental data. The maximum relative error was less than 5%.

It should be noted that, using the ROM, a significant scatter between the limit values was obtained. This means that this method should not be used to predict the fracture toughness of composites. As for the cumulative fracture probability, its values coincided with those identified for homogeneous materials [46] (the maximum difference was around 3.6%).

### 3.2. The Influence of the Effective Elastic–Plastic Parameters of the Composite on Its Resistance to Fracture

It is noted that the fracture toughness of homogeneous materials can be related to the yield stress. In the case of steels and aluminium alloys, the application of an appropriate heat treatment resulted in an increase of the yield stress value with a decrease in its fracture toughness [63]. Using the developed probabilistic model, it was verified whether the above relationship also existed in the composite materials.

**Figure 7:**
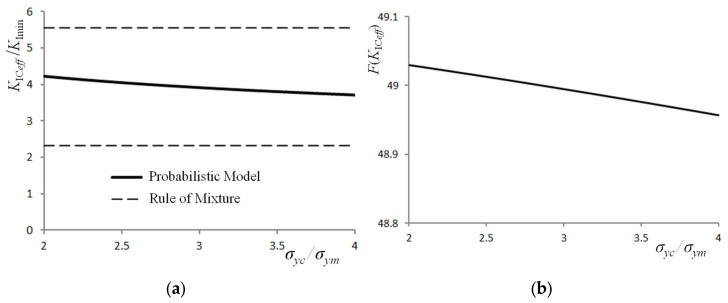
Effect of the change in the yield stress of the composite on fracture toughness (**a**) and the cumulative probability of fracture (**b**), *K_Ici_*/ *K_Icm_* = 1/8, *K*_Imin_ = *K_Ici_* = 5 (MPa·m^0.5^), *E_c_*/*E_m_* = 2, *E_m_* = 70 (GPa), *σ_ym_* = 150 (MPa), *V_i_* = 0.35.

**Figure 8:**
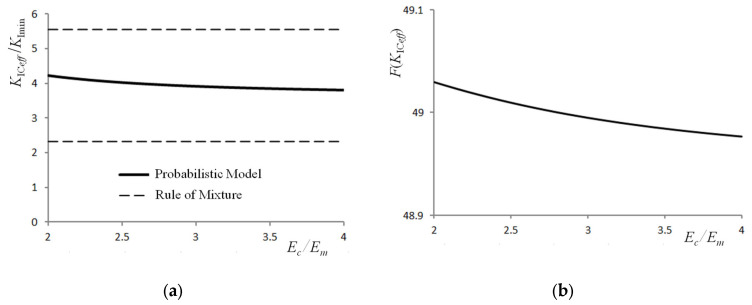
Effect of the change in the Young’s modulus of the composite on the fracture toughness (**a**) and the cumulative probability of fracture (**b**), *K_Ici_*/ *K_Icm_* = 1/8, *K*_Imin_ = *K_Ici_* = 5 (MPa·m^0.5^), *σ_yc_*/*σ_ym_* = 2, *E_m_* = 70 (GPa), *σ_ym_* = 150 (MPa), *V_i_* = 0.35.

Finally, *K*_IC*eff*_ was determined for a composite with arbitrarily assumed mechanical parameters of the reinforcement and matrix—*E_m_*, *σ_ym_*, *K_Icm_*, *K_Ici_*, *V_i_* = *constant values*—for different values of the ratios *σ_yc_*/*σ_ym_* and *E_c_*/*E_m_*. It should be noted that the effective elastic–plastic parameters (*σ_yc_*, *E_c_*) of the composite, despite the assumption of the invariability of the material parameters of the matrix and reinforcement (*E_m_*, *σ_ym_*, *K_Icm_*, *K_Ici_* = *constant values*), can take on different values. This is due to the fact that they are influenced by the microstructure of the composite, determined by factors such as, for example, the manufacturing methods and the shape and particle size of the reinforcement [64]. The obtained results are shown in Figure 7 and Figure 8. They (Figure 7a) enable concluding the following: the fracture toughness *K*_IC*eff*_, determined using the developed probabilistic model, is dependent on the yield stress *σ_yc_*. This follows the experimental results for both homogeneous materials [63] and composites [11]. In the case of the cumulative fracture probability (Figure 7b), it can be assumed that a change in *σ_yc_* has virtually no effect on the value of *F*(*K*_IC*eff*_) (a change of the order of about 1‰).

Analysing the results shown in Figure 8, it can be seen that an increase in the Young’s modulus of the composite affects the parameters tested (*K*_IC*eff*_ and *F*(*K*_IC*eff*_)) in the same way as a change in its yield stress.

In summary, all the treatments, related to the choice of manufacturing and the particle/ whisker geometry of the reinforcement fraction, aimed at achieving higher values of *σ_yc_* and *E_c_*, resulted in the simultaneous reduction of the effective fracture toughness of the composite. This may be due to the fact that any strengthening mechanism that increases the yield stress simultaneously reduces the plastic deformation capacity and, thus, the ability to blunt the tips of potential microcracks, which directly affects the effective fracture toughness of the composite. When the Young’s modulus of a material increases, an increase in its stiffness and resistance to elastic deformation is usually observed. At the same time, however, due to the reduced ability to absorb plastic deformation energy, an increase in Young’s modulus can lead to a reduction in the material’s fracture toughness, especially in the case of brittle materials.

## 4. Summary

The probabilistic model for predicting the effective fracture toughness can be used for metal matrix composites, i.e., the 44200 aluminium alloy with Al_2_O_3_ reinforcement in the form of fibres. This can be reached by means of a proprietary cumulative probability function and using a Weibull distribution. The prediction should be conducted by covering the elastic–plastic material parameters of the composite and matrix (Young’s modulus, yield stress) and the fracture toughness of the individual components of the composite considered.

In order to validate the model, the effective fracture toughness is recommended to be determined with it for several composites in which the matrix was made of different grades of aluminium alloy and the reinforcement fractions were represented by ceramic particles or short fibres. This approach enabled capturing a low value of the maximum relative error below 5%.

The influence of the elastic–plastic material parameters of the composite on its effective *K*_IC*eff*_ fracture toughness was a stage taken in the analysis. It was noted that as the Young’s modulus *E_c_* and yield stress *σ_yc_* of the composite increased, *K*_IC*eff*_ decreased with the increasing ratio of yield stress of the composite to the yield stress of the matrix.

It was also found that, at crack initiation, the cumulative probability of fracture *F*(*K*_IC*eff*_) was about 49%.

The proposed approach for determining *K*_IC*eff*_ used the global elastic–plastic parameters of the composite *σ_yc_* and *E_c_*. These parameters can be determined in an experimental approach. However, this would require a significant amount of effort and cost. Therefore, this indicated elaborating an alternative method to the experiment, which would enable *σ_yc_* and *E_c_* to be determined from the mechanical properties of the homogeneous material phases forming the composite.

## Figures and Tables

**Table 1:** Advantages, disadvantages, important results, as well as applications from the fracture toughness tests of the 44200 aluminium alloy reinforced by Saffil fibres.

Advantages	Disadvantages	Important Results	Application
The same testing machines and extensometers as for steels’ examination	U-shaped components of a gripping system should be manufactured for the specimen width	No identified fatigue pre-crack range	Modelling of MMC materials and elements with respect to fracture toughness
CTOD extensometer can be easily used, i.e., at edges in the specimen type, as well as cemented ones	Difficulties in the machining process of CT specimen	A lack of fatigue fringes	Expertise on MMC elements
Optical and microscopic devices of a typical measuring class	Low resistance to cracking	Reinforcement degradation is represented by brittle cracking	Manufacturing improvement of MMC materials
Commercial software for pre-cracking process and testing under monotonically increasing force for fracture toughness determination	Probability of crack growth in the pre-cracking fatigue stage due to the high brittleness of the material	No differences in the cracking mechanisms of the fatigue pre-crack zone and tearing one	Automotive industry (engine heads, frame sub-components for crash safety)

**Table 2:** Mechanical parameters of the matrix and reinforcement materials used in the Al44200/Al_2_O_3_ composite.

Material	Matrix			Reinforcement
	*E_m_* (GPa)	*σ_ym_* (MPa)	*K_Icm_* (MPa·m^0.5^)	*K_Ici_* (MPa·m^0.5^)
Al44200/Al_2_O_3_	72 [56]	102 [57]	12.1 [58]	4.5 [59]

**Table 3:** Material properties of the Al44200/Al_2_O_3_ composite.

Material	*V_i_* (%)	*E_c_* (GPa)	*σ_yc_* (MPa)	*K*_ICd_	*K*_IC*eff*_	*K_u_*	*K_l_*	Δ*K* (%)	*F*(*K*_IC*eff*_) (%)
				(MPa·m^0.5^)					
Al44200/Al_2_O_3_ (15%)	15	87.8	160	11.095	11.315	10.96	9.65	1.98	49
Al44200/Al_2_O_3_ (20%)	20	93.92	168	10.287	10.498	10.58	9.04	2.05	48.4
$\Delta K=\left|\frac{K_{\mathrm{ICd}}-K_{\mathrm{IC}eff}}{K_{\mathrm{ICd}}}\right|\cdot 100\%\;$									

**Table 4:** Mechanical parameters of matrix and reinforcement materials used in MMCs.

Material	Matrix			Reinforcement
	*E_m_* (GPa)	*σ_ym_* (MPa)	*K_Icm_* (MPa·m^0.5^)	*K_Ici_* (MPa·m^0.5^)
Al2124/SiC_p_	70.2 [40]	159 [40]	28.4 [40]	4.6 [60,61]
Al6061/Al_2_O_3p_	68.9 [40]	100 [40]	30 [40]	4.5 [59]

**Table 5:** Material properties of MMCs.

Material	*V_i_* (%)	*E_c_* (GPa)	*σ_yc_* (MPa)	*K*_ICd_	*K*_IC*eff*_	*K_u_*	*K_l_*	Δ*K* (%)	*F*(*K*_IC*eff*_) (%)
				(MPa·m^0.5^)					
Al2124/SiC_p_ (17%)	17	101 [40]	181 [40]	21.2 [40]	20.84	24.35	15.1	1.68	49
Al2124/SiC_p_ (25%)	25	115.8 [40]	272 [40]	18.3 [40]	17.62	22.45	12.38	3.68	48.9
Al2124/SiC_p_ (35%)	35	135.5 [40]	395 [40]	14.3 [40]	14.98	20.07	10.1	4.75	46.4
Al6061/Al_2_O_3p_ (15%)	15	90 [62]	338 [62]	18 [15]	18.76	26.17	16.21	4.23	49
$\Delta K=\left|\frac{K_{\mathrm{ICd}}-K_{\mathrm{IC}eff}}{K_{\mathrm{ICd}}}\right|\cdot 100\%\;$									

## Data Availability

The data presented in this study are available upon request from the corresponding author. At the time the project was carried out, there was no obligation to make the data publicly available.
